# Dispersal and genetic structure in a tropical small mammal, the Bornean tree shrew (*Tupaia longipes*), in a fragmented landscape along the Kinabatangan River, Sabah, Malaysia

**DOI:** 10.1186/s12863-020-00849-z

**Published:** 2020-04-17

**Authors:** Jennifer Brunke, Isa-Rita M. Russo, Pablo Orozco-terWengel, Elke Zimmermann, Michael W. Bruford, Benoit Goossens, Ute Radespiel

**Affiliations:** 1 University of Veterinary Medicine Hannover, Institute of Zoology, Buenteweg 17, 30559 Hannover, Germany; 2 Organisms and Environment Division, Cardiff University, School of Biosciences, Sir Martin Evans Building, Museum Avenue, Cardiff, CF10 3AX UK; 3 Sustainable Places Research Institute, Cardiff University, 33 Park Place, Cardiff, CF10 3BA UK; 4 Sabah Wildlife Department, Wisma Muis, 88100 Kota Kinabalu, Sabah Malaysia; 5 Danau Girang Field Centre, c/o Sabah Wildlife Department, Wisma Muis, 88100 Kota Kinabalu, Sabah Malaysia

**Keywords:** Forest fragmentation, Migration, *Tupaia longipes*, Borneo, Microsatellites, Cytochrome b, Population structure, Genetic differentiation, Sex-biased dispersal

## Abstract

**Background:**

Constraints in migratory capabilities, such as the disruption of gene flow and genetic connectivity caused by habitat fragmentation, are known to affect genetic diversity and the long-term persistence of populations. Although negative population trends due to ongoing forest loss are widespread, the consequence of habitat fragmentation on genetic diversity, gene flow and genetic structure has rarely been investigated in Bornean small mammals. To fill this gap in knowledge, we used nuclear and mitochondrial DNA markers to assess genetic diversity, gene flow and the genetic structure in the Bornean tree shrew, *Tupaia longipes,* that inhabits forest fragments of the Lower Kinabatangan Wildlife Sanctuary, Sabah. Furthermore, we used these markers to assess dispersal regimes in male and female *T. longipes.*

**Results:**

In addition to the Kinabatangan River, a known barrier for dispersal in tree shrews, the heterogeneous landscape along the riverbanks affected the genetic structure in this species. Specifically, while in larger connected forest fragments along the northern riverbank genetic connectivity was relatively undisturbed, patterns of genetic differentiation and the distribution of mitochondrial haplotypes in a local scale indicated reduced migration on the strongly fragmented southern riverside. Especially, oil palm plantations seem to negatively affect dispersal in *T. longipes.* Clear sex-biased dispersal was not detected based on relatedness, assignment tests, and haplotype diversity.

**Conclusion:**

This study revealed the importance of landscape connectivity to maintain migration and gene flow between fragmented populations, and to ensure the long-term persistence of species in anthropogenically disturbed landscapes.

## Background

Deforestation of tropical rainforests causes severe problems for the maintenance of biodiversity and ecological functions worldwide [1–3]. Agricultural expansion, logging, and urbanisation often results in a matrix of altered terrain surrounding original habitat patches. Landscape matrix features and inherent ecological and behavioural plasticity determine the permeability of the surrounding landscapes for a given species [3, 4]. Species unable to penetrate the surrounding matrix may experience limits to their movement between habitat patches. Restricted migration between populations results in a reduction in gene flow and connectivity which may lead to a decrease in viability and persistence of isolated populations [3, 5]. However, the extent to which habitat fragmentation has a negative effect on the genetic structure and persistence of animal populations remains debated [6]. While some studies have demonstrated a pronounced effect of habitat fragmentation on species’ population genetic structure (e.g., [7–12]), others have failed to detect these effects (e.g., [11, 13–15]). In this context, a better understanding of how and the degree to which fragmentation affects species will help to understand demographic processes that are triggered by fragmentation events.

One of the highest rates of deforestation in the tropics has occurred on Borneo [2]. Much of the original large forested areas on Borneo have already been lost due to logging or forest conversion to agriculture [16, 17]. The remaining forests are restricted to isolated patches in many locations. Along the Kinabatangan River of north-eastern Borneo, much of the original lowland dipterocarp forest was cleared or converted within the last 30–40 years [9, 18]. Nowadays, oil palm plantations dominate the landscape along the river and forest is restricted to isolated patches. Despite this extensive land conversion in the Kinabatangan floodplain, a highly diverse small mammal community remains in the forest remnants along the Kinabatangan River [19]. Diverse responses to landscape features along the Kinabatangan River itself have recently been reported for a suite of small mammals in this anthropogenically modified region [19]. Dispersal and gene flow have been shown to be limited by the Kinabatangan River predominantly in squirrels and tree shrews. However, signals of high genetic differentiation between populations have suggested additional limitations in terrestrial vagility along riverbanks for these taxa. The consequences of habitat fragmentation on dispersal and genetic diversity are still largely unknown for Bornean non-volant small mammals. The relative scarcity of systematic data on the effects of fragmentation in Bornean small mammals is alarming, as they provide important ecosystem functions and services for their natural habitats [20]. They are important seed dispersers, pollinators, invertebrate and seed predators, and are prey for larger predators [20–23].

Along the Kinabatangan River, habitat connectivity differs considerably on both riversides. While forest connectivity is high on the northern riverbank, it is disrupted (mainly by oil palm plantations) on the southern side. This setting provides a heterogeneous landscape highly suitable for estimating and comparing migratory activities and genetic structures under different habitat configurations.

The Bornean tree shrew (*Tupaia longipes* Thomas 1893, Scandentia, Tupaiidae) is endemic to Borneo, is widespread throughout the island and is known to inhabit fragmented forest patches such as those along the Kinabatangan River [19]. Although listed by the IUCN as *Least Concern* [24], *T. longipes* has a declining population trend throughout its range due to deforestation and habitat degradation, and is therefore listed in CITES Appendix II [25]. It is restricted to habitats with dense understory, and occurs in virgin forest, logged forest [26], but also in tree plantations with dense understory [27, 28]. In habitats without dense understory *T. longipes* is largely absent [29]. Being a terrestrial surface gleaner, it feeds mainly on insects and other arthropods, but also on fruits. It is a very agile tree shrew species with large home ranges and large daily travel distances (up to 2 km) compared to other tree shrews [21]. Based on behavioural observations, a facultative monogamy is the dominant social system suggested for tree shrews, including *T. longipes* [21, 30–32]. Various olfactory and acoustic signals govern their social interactions [33, 34]. Although female-biased dispersal is known from other tree shrew species [35], and Wells et al. [36] suggested that sex-biased dispersal may be found in a variety of Bornean small mammals including *T. longipes*, no information exist on its dispersal regime so far.

**Fig. 1:**
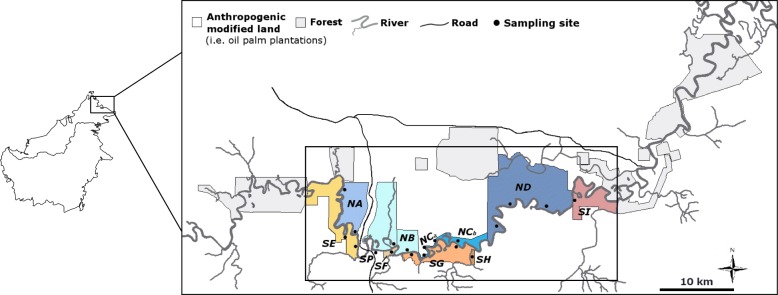
Map of the Lower Kinabatangan Wildlife Sanctuary (LKWS) showing the distribution of sampling locations (circles) and respective forest sites (coloration). The smaller black square highlights the study area within the LKWS

Here we analysed and compared nuclear and mitochondrial genetic diversity and genetic structure in *T. longipes* subpopulations from both sides of the Kinabatangan River (Fig. 1) to assess constraints in migration and gene flow due to relatively recent habitat fragmentation. If this fragmentation limits migration in *T. longipes*, genetic discontinuities and genetic differentiation should be higher in landscapes comprising isolated forest fragments, compared to those that are contiguous and uniform. Furthermore, habitat alterations may affect dispersal of males and females differently, if they possess sex-biased dispersal [37]. We therefore investigated sex-specific dispersal in males and females.

By identifying potential disruptions in gene flow, we will compare the importance of landscape modifications for generating genetic structures and will provide knowledge for the development of effective conservation measures and landscape management plans that help to improve population connectivity in anthropogenically disturbed landscapes.

## Results

### Genetic diversity and population structure along the Kinabatangan River

Individuals of *T. longipes* were present in all except one forest site (site SH), and were absent in the plantation site (site SP, Fig. 1). These sites were thus not included in the following analyses.

All eight loci (Additional file 1, Table S1) were polymorphic, but null alleles were present in locus TB 14. The number of alleles per locus varied between 4 and 36 and allelic richness between 2.670 and 7.115 (Table 1). Locus TB 14 had a significant Hardy-Weinberg equilibrium (HWE) departure across the whole dataset and most forest sites and thus was removed from further analyses (Table 1). A significant departure from HWE was present in locus TB 8 for site NA and site ND, however, as no overall HWE departure was observed this locus was kept. While a significant overall departure from HWE could be detected in locus TB 18, HWE-departures were not evident at individual forest sites for this locus, hence it was retained. Testing for Linkage disequilibrium (LD) with Bonferroni correction, seven pairwise comparisons were significant but with no consistent pattern across remaining loci.

**Table 1:** Genetic characteristics of analysed loci (upper half) and sites (lower half). Number of alleles per locus (N_a_), the size range of each locus, allelic richness, unbiased expected heterozygosity (H_e_), observed heterozygosity (H_o_), fixation index (F_IS_), deviations from HW equilibrium, total number of analysed samples (n), number of males and females, number of sequenced samples and haplotypes, haplotype diversity (*h*) and nucleotide diversity (*π*) for each site and riverside, respectively

Locus	N_a_	Size range [bp]	Allelic richness	H_e_	H_o_	F_IS_	Sites out of HWE			
Js 22	7	172–186	2.670	0.496	0.483	0.027				
Js 183	11	134–144	4.373	0.794	0.759	0.044				
Js 188	15	182–201	4.542	0.789	0.759	0.039				
TB 8	4	404–420	2.831	0.621	0.595	0.043	NA, ND			
TB 14	36	457–569	7.115	0.966	0.482	0.503**	all, exc. SI			
TB 15	31	284–344	6.601	0.942	0.905	0.040				
TB 16	16	170–203	4.174	0.697	0.655	0.060				
TB 18	32	408–544	6.452	0.933	0.888	0.049*				
Site	n	Males/Females	Allelic richness	H_e_	H_o_	F_IS_	Sequences/Haplotypes	*h*	*π*	Area [km^2^]
NA	11	6 / 5	4.911	0.725	0.727	−0.004	8 / 6	0.893	0.008	22.17
NB	18	10 / 8	5.105	0.788	0.786	0.003	7 / 4	0.714	0.007	41.60
NC	5	4 / 1	4.571	0.698	0.771	−0.119	2 / 2	1.000	0.012	9.66
ND	17	9 / 8	5.013	0.731	0.714	0.024	10 / 5	0.800	0.008	73.17
North	51	29 / 22	4.962	0.761	0.748	0.017	27 / 11	0.889	0.009	146.60
SE	11	8 / 3	4.257	0.740	0.753	−0.019	6 / 3	0.733	0.005	47.99
SF	21	9 / 12	3.902	0.685	0.674	0.018	7 / 4	0.810	0.005	1.25
SG	29^a^	14 / 14	3.888	0.689	0.714	−0.037	17 / 3	0.581	0.004	17.14
SI	4	3 / 1	3.286	0.628	0.571	0.103	3 / 2	0.667	0.012	16.14
South	65^a^	34 / 30	3.895	0.718	0.699	0.026	33 / 6	0.695	0.006	82.52
All	116^a^	63 / 52	4.414	0.753	0.720	0.044**	60 / 14	0.878	0.008	229.12

*p ≤ 0.05, **p ≤ 0.01, ^a^one individual of unknown sex

The values of observed (H_o_) and expected heterozygosity (H_e_) as well as the inbreeding coefficient (F_IS_) were not significantly different between riverbanks (Table 1; N_north_ = 4, N_south_ = 4; H_o_: *p* = 0.110; H_e_: *p* = 0.149; F_IS_: *p* = 0.773), while allelic richness was significantly lower south of the river (N_north_ = 4, N_south_ = 4, *p* = 0.021; Table 1).

Across the 60 mitochondrial cytochrome b (*cyt b*) sequences (693 bp length), 28 variable sites (23 transitions, 5 transversions) and 14 haplotypes were identified. Haplotype diversity (*h*) ranged from 0.587 to 1.000, and nucleotide diversity (*π*) from 0.004 to 0.012 (Table 1). Neither haplotype (N_north_ = 4, N_south_ = 4; *p* = 0.149) nor nucleotide diversity (N_north_ = 4, N_south_ = 4; *p* = 0.186) differed significantly between the two riversides (Table 1).

F_ST_-values varied between 0.0040 (between site NA and ND) to 0.0847 (between site ND and SE) and most were significant (*N* = 15, mean F_ST_ = 0.0445; Additional file 1, Table S2). On average, F_ST_-values were higher between sites located on different riverbanks (*N* = 9, mean F_ST_ = 0.0575) than on the same riverside (*N* = 6, mean F_ST_ = 0.0249), with, F_ST_-values between southern sites (*N* = 3; F_ST_ = 0.0353) higher than between northern sites (*N* = 3, mean F_ST_ = 0.0144; *p* = 0.050; Additional file 1, Table S2 and S3).

A similar pattern was found in the mean relatedness between forest sites, with values ranging from − 0.1054 (between site ND and SE) to 0.0532 (between site NA and ND; Additional file 1, Table S2). Overall no relatedness exists between the 5671 analysed dyads (*r* = − 0.0021), and between dyads from different riversides (*N* = 2806, *r* = − 0.0467), but mean relatedness was relatively high within riversides (*N* = 1015, *r* = 0.0416). Relatedness was higher in the southern (*N* = 1830, *r* = 0.0616) than in the northern subset (*N* = 1035, *r* = 0.0064; *p* < 0.001; Additional file 1, Table S2 and Table S3). In particular, within southern forest sites a high mean relatedness was found between dyads (N_north_ = 344, *r*_north_ = 0.0247; N_south_ = 671, *r*_south_ = 0.1113; *p* < 0.001), but also among forest sites mean relatedness was higher in the south (*N* = 1159, *r* = 0.0329) than in the north (*N* = 691, *r* = − 0.0028; *p* < 0.001; Additional file 1, Table S2 and Table S3).

Although the low number of sites along each riverbank prevented an unbiased isolation-by-distance analysis, no obvious correlation between pairwise genetic differentiation (F_ST_) or mean relatedness and geographic (Euclidian) distance was observed on either riverbank (Additional file 1, Table S2). However, northern riverside F_ST_-values were lower and mean relatedness was higher between the most remote sites (site NA and ND), while the opposite was observed between the most distant southern forest sites (site SE and SG*;* Additional file 1, Table S2).

**Fig. 2:**
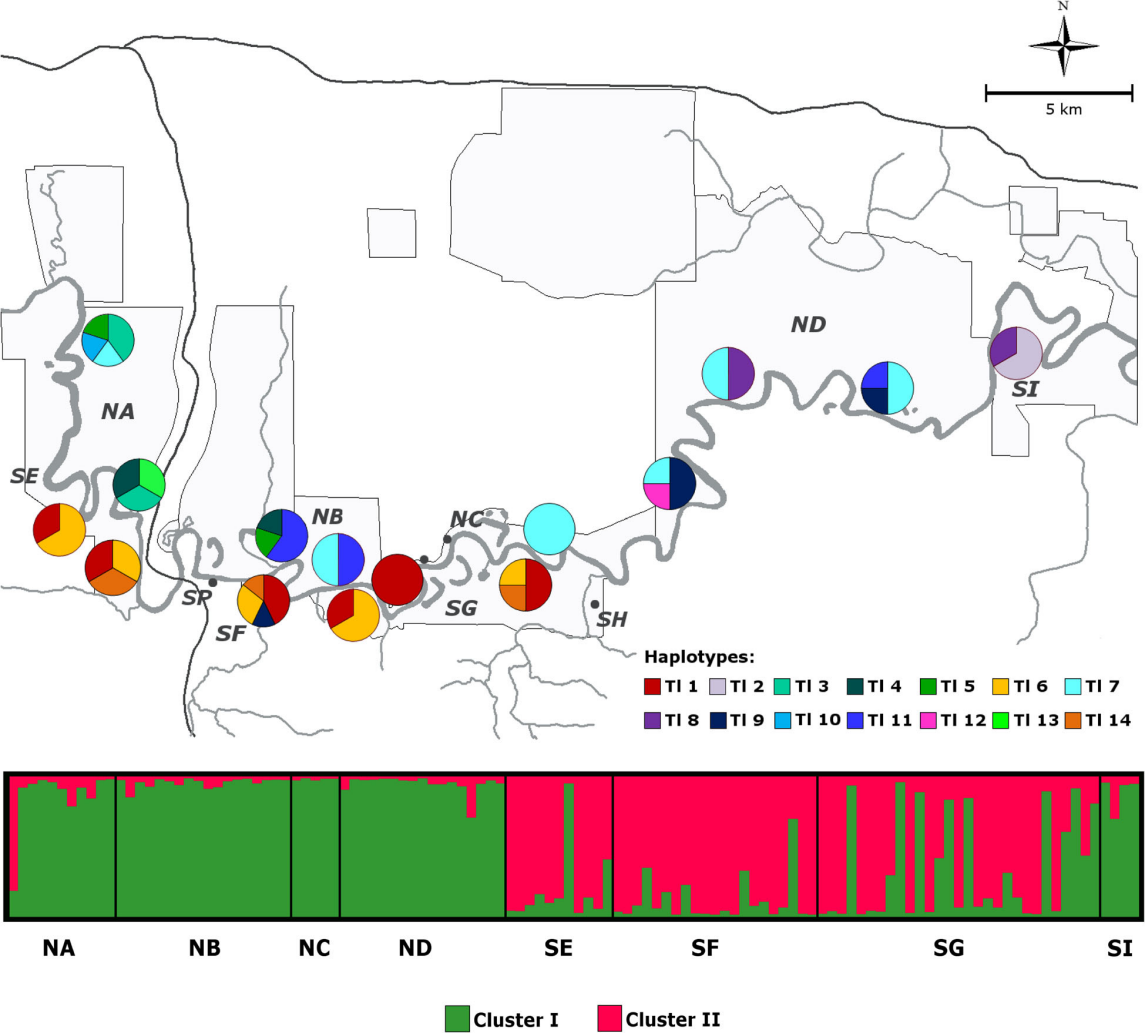
Spatial distribution of mitochondrial *cyt b* haplotypes (above) and Bayesian *STRUCTURE* plot (below) showing the membership of individuals for k = 2 clusters (based on nuclear microsatellite genotypes)

*STRUCTURE* analysis suggested the existence of two population clusters within *T. longipes* with the highest likelihood (LnP(D): − 3112.2; Fig. 2). As the method of Evanno et al. [38] is prone to produce biases toward *k* = 2 [39], the next highest Δk values at *k* = 3 and *k* = 6 were also considered. However, both showed no further geographically meaningful clustering of individuals. At *k* = 2 (used for further analyses) 103 out of 116 individuals could be assigned to one particular cluster with a *q* > 80% probability (Additional file 1, Table S4). Individuals from the northern riverside were almost exclusively assigned to cluster I. On the southern riverside individuals from sites upstream were mostly assigned to cluster II, while those from sites downstream were assigned to either cluster (Fig. 2, Additional file 1, Table S4). A progressive partitioning approach for the northern and southern sample subsets yielded solely two sub-clusters in the southern subset. However, these provided no further geographically meaningful information.

Among the 14 mitochondrial haplotypes, 11 were found in samples from the northern side and only six occurred in the southern subset (Table 1, Additional file 2, Table S5). The haplotype network revealed only three haplotypes (Tl 1, Tl 8, Tl 9) that were shared between riversides (Fig. 2, Additional file 2, Fig. S1). On the northern riverside one haplotype (Tl 7) was shared between all sites and another haplotype (Tl 11) occurred in spatially distant sites (sites NB and ND, Fig. 2). On the southern riverside three haplotypes (Tl 1, Tl 6, Tl 14) were shared between the adjacent sites SE, SF, and SG, but did not occur in the more remote site SI. Conversely, two haplotypes (Tl 2, Tl 8) were identified in site SI which occurred nowhere else on the southern riverside (Fig. 2).

### Migration and gene flow across and along the Kinabatangan River

Results obtained with BayesASS indicated a very low proportion of individuals migrating per generation (about one individual per generation in either direction) between riversides (from south to north: 0.0161 ± 0.0137; from north to south: 0.0092 ± 0.0084). Within riversides, mean migration rates among northern forest sites was 0.0617 (± 0.0221), and on the southern riverside 0.0531 (± 0.0364). However, migration rates were not evenly distributed among sites. On the northern riverside, high migration rates (> 0.15) were calculated for site NB to all other sites (Table 2). On the other hand, site NB had the highest proportion of residents among all sites and thus did not receive many immigrants (Table 2). On the southern riverside the sites SF and SG were the sources of most individuals migrating to other sites with SE and SI being the main acceptors from these fragments, respectively (Table 2).

**Table 2:** Migration rates between pairs of forest sites from the northern (NA–ND) and southern (SE–SI) riverside. The values for each forest site (row) are the proportion of migrants (± SD) deriving from another site (column). Migration values ≥0.10 are highlighted in bold

	Migrants								
	NA	NB	NC	ND	SE	SF	SG	SI	Residents
NA		**0.2014**	0.0171	0.0180	0.0185	0.0221	0.0209	0.0175	**0.6845**
		± 0.0383	± 0.0164	± 0.0170	± 0.0174	± 0.0204	± 0.0196	± 0.0166	± 0.0169
NB	0.0135		0.0135	0.0148	0.0141	0.0172	0.0386	0.0136	**0.8746**
	± 0.0128		± 0.0129	± 0.0151	± 0.0135	± 0.0159	± 0.0361	± 0.0129	± 0.0436
NC	0.0261	**0.1526**		0.0253	0.0255	0.0258	0.0271	0.0255	**0.6921**
	± 0.0245	± 0.0452		± 0.0236	± 0.0238	± 0.0235	± 0.0249	± 0.0236	± 0.0236
ND	0.0133	**0.2309**	0.0133		0.0140	0.0151	0.0177	0.0133	**0.6823**
	± 0.0129	± 0.0337	± 0.0127		± 0.0132	± 0.0150	± 0.0196	± 0.0126	± 0.0164
SE	0.0181	0.0233	0.0178	0.0174		**0.1509**	0.0689	0.0179	**0.6857**
	± 0.0170	± 0.0213	± 0.0172	± 0.0164		± 0.0619	± 0.0566	± 0.0170	± 0.0180
SF	0.0118	0.0163	0.0117	0.0122	0.0126		0.0737	0.0113	**0.8505**
	± 0.0114	± 0.0155	± 0.0112	± 0.0117	± 0.0121		± 0.0747	± 0.0110	± 0.0760
SG	0.0093	0.0153	0.0093	0.0092	0.0098	**0.1065**		0.0092	**0.8313**
	± 0.0091	± 0.0144	± 0.0091	± 0.0091	± 0.0095	± 0.0831		± 0.0089	± 0.0824
SI	0.0277	0.0455	0.0277	0.0278	0.0279	0.0298	**0.1189**		**0.6946**
	± 0.0258	± 0.0348	± 0.0253	± 0.0256	± 0.0258	± 0.0292	± 0.0476		± 0.0259

### Reconstruction of dispersal in males and females

Sex-specific relatedness was assessed in a total of 1953 male/male dyads and 1326 female/female dyads. The overall mean relatedness was low in both sexes. However, while within sampling locations no difference in mean relatedness exists between males and females, among sampling locations (within riversides) higher relatedness exists in male than in female dyads (Table 3). The overall number of related dyads (*r* ≥ 0.25) was similar for males (*N* = 105, 5.4%) and females (*N* = 91, 6.9%), but related males were spatially more distant than related females. This pattern was also present within riversides when comparing related males and females from different sampling locations (Table 3).

**Table 3:** Relatedness (*r*, mean ± SD), amount of related dyads (*r* ≥ 0.25) and interindividual distance (mean ± SD) for all related male and female pairwise comparisons. Within riversides results of within and among sampling location comparisons are given. The mean (*mAIc* ± SD) and variance (*vAIc*) of corrected assignment indices are given for the overall male and female subset. Parameters differing significantly between sexes are given in bold (details of statistical tests are in Additional file 1, Table S3)

	Males						Females					
	No. of dyads	r	related dyads [%]	distance [km]	mAIc	vAIc	No. of dyads	r	related dyads [%]	distance [km]	mAIc	vAIc
All	1953	−0.0032^**^ ±0.1885	5.38	7.59^**^ ±7.92	0.0310 ±1.6967	2.8790^**^	1326	−0.0205^**^ ±0.1942	6.86	4.24^**^ ±5.94	−0.0419 ±2.4774	6.1375^**^
within riversides, within sampling locations	94	0.1187 ±0.2101	23.40	0.06 ±0.05			81	0.1302 ±0.2250	27.16	0.06 ±0.05		
within riversides, among sampling locations	873	0.0407^**^ ±0.1811	7.67	8.82^*^ ±7.65			585	0.0130^**^ ±0.1977	9.23	4.95^*^ ±5.93		

* *p* ≤ 0.05, **p ≤ 0.01

Although no significant differences were detected in overall *mAIc* of males and females in the assignment tests (Table 3), the overall male *mAIc* was positive, suggesting higher proportions of male residents, while the overall female *mAIc* was negative, indicating higher proportions of female immigrants. In accordance with their negative *mAIc*, female *AIc* values showed a higher variance than those of males (Table 3).

In a subsample of 28 males and 31 females mitochondrial haplotype diversity was assessed as a further determinant of sex-biased dispersal. Although the overall number of haplotypes (males = 11, females = 13), and the number of sex-specific haplotypes (males = 1, females = 3), were slightly higher in females, the number of unique haplotypes at a given site (*haplotype singletons*: males = 11, females = 10) was rather evenly distributed between sexes, and no clear sex-specific dispersal pattern could be inferred.

## Discussion

### The influence of the Kinabatangan River and the landscape on genetic diversity and structure

The present study corroborates some findings of Brunke et al. [19] and confirms the river as an important barrier to gene flow in *T. longipes*. For example, when ignoring the river by pooling the northern and southern samples, a significant excess of homozygosity was visible in the whole dataset, most likely a result of the Wahlund effect [40]. Since this effect is absent within the northern and southern subsamples, *T. longipes* from the two riversides of the Kinabatangan River can be assumed to belong to two genetically distinct sub-populations. This assumption is further supported by the results of the *STRUCTURE* analysis, in which samples were allocated to two population clusters, with the two clusters largely representing one riverside each. However, the migration rates assessed with *BayesASS*, the sharing of three haplotypes between riversides and the allocation of some southern individuals to the northern population cluster indicate some degree of genetic exchange between the two riverside subpopulations. Occasional crossings of the river are therefore likely to occur.

Genetic differentiation among populations can also be generated without geographic isolation, for example, as a result of past colonization processes [41]. The observed patterns of high mtDNA haplotype and low nucleotide diversity suggest an influence of such processes on the population structure in *T. longipes* [42]. Most likely, historical habitat contractions and fragmentation during Pleistocene glaciation, and a later colonization of the Kinabatangan floodplain from glacial refuges has shaped such signals in *T. longipes*, as proposed for many other animal and plant species of this region (e.g., [43–46]). In addition to historical population processes, recent anthropogenic modification (i.e. forest conversion to oil palm plantations) in this region markedly shaped the landscape within the last 30–40 years [9, 18]. The shaped landscape differed considerably between riversides. While on the northern side of the Kinabatangan River forests are still connected by a forest corridor, the forest fragments on the southern riverbank are separated mainly by oil palm plantations. Considerable differences in the remaining forest size within the study area (north = 147 km^2^, south = 83 km^2^; Table 1) further underline the stronger fragmentation of forests in the south. Considering the short generation time of 1–2 years in tree shrews with 1–2 litter per year (sometimes three, in times of supermasting [21, 47]), genetic patterns in the studied *T. longipes* population may already reflect this recent landscape fragmentation. For example, low F_ST_-values, high relatedness and shared haplotypes suggested ongoing gene flow even between distant sites in the contiguous forest on the northern riverside and less in the fragmented southern forest. However, migration rates assessed with BayesASS suggested some (between some forest sites rather low) gene flow between study sites on both riversides. On the northern riverside, site NB seems to act as an important source of immigrants to the other northern sites, possibly because of its central position. Although less pronounced than on the northern riverside, migration also took place between sites on the southern riverbank. Again, migrants originated predominantly from central sites (i.e., site SF and SG).

A triangulation study of *T. longipes,* which was carried out on two animals in one forest fragment along the Kinabatangan River (forest site SG), revealed home range sizes of 13–16 ha (Brunke et al. unpublished data, Additional file 3, Table S6). Furthermore, high distances traveled per day (~ 2 km) are known for this species [21]. Bowman et al. [48] showed that home range size co-varies with maximum dispersal distances in mammals. Based on the underlying mathematical relationship, a maximum dispersal distance of about 16 km could be suggested for *T. longipes*. In our study, neighboring sites within each riverside were 6–22 km (Additional file 1, Table S2) apart from each other and therefore ranged within this potential maximum dispersal distance of *T. longipes*. This together with a rather low sample size might be reasons why no obvious isolation-by-distance effect (although not explicitly tested) could be detected on both sides of the river.

On the other hand, the signals of decreased connectivity and genetic diversity on the southern riverside may be interpreted as a consequence of more intense and/or earlier landscape fragmentation on the southern side of the river. For example, it cannot be finally decided whether migration between distant sites in the north is still ongoing under the current degree of fragmentation, or if the inferred migration rates may be partly a signal reflecting pre-fragmentation connectivity. While the southern forest sites already started to get fragmented by ongoing landscape modifications (i.e. expansion of oil palm plantations) in early 1980s, forests on the northern side largely retained their connectivity until mid to late 1990s (Additional file 3, Figure S2). This longer period of forest connectivity north of the river may also explain the lower genetic differentiation among the northern sites.

Following the principles of the coalescence theory, high frequency haplotypes represent old alleles, while haplotype singletons and haplotypes with low frequency are likely to have evolved rather recently [40]. Moreover, “old” haplotypes are expected to show a broader spatial distribution than more recent ones, because their carriers had longer times to disperse [40]. The comparison of haplotype frequencies and the spatial distribution patterns of haplotypes on both riversides indeed suggest longer forest connectivity in the north. Conversely, the earlier fragmentation processes on the southern riverside may have shaped genetic structure by a relatively early reduction of migration between forest patches, resulting in higher genetic differentiation between sites, a lower number of haplotypes, and in an accumulation of “older” haplotypes on this side of the river.

In addition, the presence of unique haplotypes in the isolated site SI (and the absence of these haplotypes in other southern sites), the high genetic relatedness within forest sites, and the heterogeneous representation of genetic clusters on the southern side suggests that plantations have further reduced genetic connectivity. Other potential barriers such as roads or tributaries did not seem to have influenced gene flow in *T. longipes*, possibly because they are not wide enough to affect dispersal in this species [49, 50]. These results are congruent with findings in murids of this region [19] in which signals of genetic differentiation in mtDNA sequences existed between sites separated by large plantations. Furthermore, other studies have demonstrated that *T. longipes* is absent in habitats with poor understory structures, such as oil palm plantations [29], and this was also the case in our study (site SP, Fig. 1). As it is known that *T. longipes* prefers to move in habitats with dense understory [21], a disrupting effect of oil palm plantations (in which dense understory is largely absent) on connectivity is therefore highly likely.

### Dispersal of males and females along the Kinabatangan River

Sex-biased dispersal should be reflected in the relatedness structure within populations because the dispersing sex will transfer alleles that do not correspond to the local allele distribution and thus can be identified by a lower overall relatedness compared to the more philopatric sex [51]. Overall relatedness was low in both sexes, but a higher mean relatedness in males than in females from different locations, suggesting female-biased dispersal in *T. longipes*. In contradiction, however, suggest the higher distance of related males a farther reaching dispersal in males. The results of the assignment test were also ambiguous: both sexes had quite low and similar *mAIc* values (= low likelihoods to be resident), whereas females had higher *vAIc* values (= more variability) which would be expected for the dispersing sex. To detect a signature of sex-biased dispersal using *mAIc,* actual differences between the sexes should be substantial (not less than 80:20, [52]). Following this guideline, *T. longipes* would have a rather unbiased dispersal system. The evenly distribution of haplotypes between sexes further support this.

*Tupaia longipes* forms strong pair bonds with a pronounced intrasexual territoriality, and female home ranges are large [21] (Brunke et al. unpublished data, Additional file 3, Table S6), males are thus not able to monopolise more than one (sometimes two) female(s). Munshi-South [35] hypothesised that the energetically-expensive absentee maternal care system, observed in tree shrews [21, 30], restricts the ability to rear young on poor-quality territories, and produces intense competition between females for resources [47]. In order to avoid competition and to ensure their own reproductive success, females may be driven to disperse from their natal site to settle in a distant, high-quality territory. Female dispersal may be driven by this pattern in *T. longipes*, whereas the farther male dispersal may be a consequence of female dispersion and intense male-male competition for access to females in *T. longipes*. However, this hypothesis needs to be explicitly tested to assess the driving factors for male and female dispersal in this species.

## Conclusion

This study demonstrates that the Kinabatangan River and other landscape features have most likely shaped the genetic structure of *T. longipes* populations in historic and in recent timescales. Moreover, it demonstrates that anthropogenic forest conversion starting 30–40 years ago [9, 18] can already be detected by increased genetic differentiation and a decrease in gene flow between populations of *T. longipes*. In particular, signals of restricted gene flow were pronounced on the strongly fragmented southern riverside, possibly as an additive effect of earlier and stronger landscape modifications (i.e. expansion oil palm plantations).

There have been contrasting results on the effectiveness of corridors to reduce effects of habitat fragmentation [53–55]. However, Bruford et al. [56] showed that the establishment of forest corridors in the Kinabatangan area can help to retain demographic stability of isolated orangutan (*Pongo pygmaeus*) sub-populations. It has been demonstrated that corridors need to be sufficiently wide to reduce the vulnerability to edge effects and increase its structural habitat heterogeneity [57, 58]. Lees and Peres [59] proposed a minimum corridor width of about 400 m to provide appropriate habitats for various species. The corridor width on the northern Kinabatangan riverbank ranged between 140 and 2000 m, and thus should serve well to maintain migration and to reduce genetic isolation between demes. Moreover, it contributes in the enlargement of effective forest expanse in the north. On the southern side a corridor is largely absent, only a few forest trees with scattered availability connect some of the southern patches along the Kinabatangan River. In our study, this seems not to be effective in providing sufficient habitat connectivity. Possibly because, corridors should reflect natural landscape features, which do not typically take the shape of narrow habitat strips [57, 60]. Although some degree of gene flow may still be maintained between the southern forest fragments up to the present, the negative effect of habitat fragmentation and isolation on genetic diversity might increase further in future generations by the ongoing agricultural expansion in this region [17, 61]. This is an alarming trend by which the long-term viability of *T. longipes* populations may become jeopardized. Considering that according to the IUCN [24] more than half of the small mammal species confirmed for this region [19] show a decline throughout their range, it is important to ensure gene flow between isolated demes that may enable them to avoid the negative effects of demographic isolation.

Our study also tried to unravel sex-specific dispersal patterns, to estimate impacts of habitat fragmentation on male and female dispersal. An overall high interindividual relatedness in the *T. longipes* sub-population could already be verified within the fragmented forests in the south, and inbreeding or kin competition may occur when small habitat patches remain isolated [37, 62]. Moreover, to avoid inbreeding or competition pressure modifications in dispersal patterns of males and females may occur with substantial long-term changes in within-population social and demographic dynamics. Whether the dispersal pattern found in males and females of *T. longipes* along the Kinabatangan River is already influenced by the fragmentation cannot be said for certain, and further comparative studies, both in connected and fully isolated habitats are clearly necessary to investigate the social and behavioral consequences of forest fragmentation and disrupted dispersal patterns in this species. An effective landscape management approach combining the preservation of forest remnants with an effective corridor planning to ensure connectivity between isolated patches will be essential to mitigate ongoing fragmentation effects and ensure long-term survival of animal populations within already altered habitats.

## Methods

### Sampling

The study was conducted in 18 forest sites and one plantation site along the Lower Kinabatangan Wildlife Sanctuary (LKWS) in eastern Sabah between 2011 and 2013 (Fig. 1). Sampling locations in close spatial proximity and without separation by a geographic feature were considered as one site for most analyses, as shown in Fig. 1. For the present study, forest sites NC_a_ and NC_b_ were pooled to site NC due to small sample sizes.

Small ear tissue biopsies (obtained from the ear pinnae) were collected from *T. longipes* individuals that were live-trapped in the LKWS. All individuals were released back to the wild at their individual capture locations after handling. A more detailed description of the study area, sites and the sampling procedure can be found in Brunke et al. [19].

Sampling and handling protocols were reviewed and approved by the Institute of Zoology, University of Veterinary Medicine Hannover, Germany, Cardiff University, UK, and the Danau Girang Field Centre, Malaysia. Official statements from ethical committees are not required under German and UK law for research carried out abroad. All field protocols reported in this study adhered to the legal requirements of Malaysia and the state of Sabah. All methods were officially approved by the Economic Planning Unit Malaysia (Permit No.: UPE:40/200/19/2871) and the Sabah Biodiversity Centre. This research also adhered to the guidelines of the American Society of Mammalogists (ASM; Animal Care and Use Committee, 2011) for the ethical handling of animals.

Samples were transported under the permits of CITES [Malaysian Export-Permit No. JHL (PB)600–3/18/1/1Jld.10/(103), Certificate No. 0602 and Export-Permit No. JHL (PB)600–3/18/1/1Jld.10/(494), Certificate No. 0689 and 0690; German Import-Permit No. E-05027/12 and E-05957/13] and the Sabah Biodiversity Centre [Export-Licence No.: JKM/MBS.1000–2/3(38)].

### Molecular methodology

DNA was extracted from *T. longipes* tissue biopsy samples from 116 individuals following a HotSHOT extraction protocol [63]. All samples were genotyped using microsatellite (*msat*) primers described by Munshi-South and Wilkinson [64], and Liu and Yao [65]. Out of 14 available primers, only eight *msat* loci were applicable on the *T. longipes* samples used in this study (Additional file 1, Table S1). All forward primers were fluorescently labeled and PCR reactions were performed in 10 μl with 5 μl Multiplex PCR Master Mix (Qiagen, Hilden, Germany), 0.1 μl Q-solution (Qiagen), 0.2 pmol of each primer, and 1 μl template DNA. DNA amplification was carried out in three multiplexes (M1–M3) and two single reactions (S1–S2; Additional file 1, Table S1) with an initial denaturation at 95 °C for 15 min, followed by 35 cycles of 45 s at 95 °C, 1:30 min at varying annealing conditions (Additional file 1, Table S1) and 1 min at 72 °C, and a final extension at 72 °C for 10 min. PCR products were analysed at Dundee Biosciences, Scotland. Allele length was determined using GeneMapper version 5.0 (Applied Biosystems, USA). Homozygote samples were reanalysed at least twice to minimize genotyping errors.

A subset of 60 samples was sequenced at the mitochondrial cytochrome b (*cyt b*) locus to support the *msat*-analyses. For the sequencing, an optimised primer (L14841tupaia) together with the primer MVZ16 was used as described in Brunke et al. [19]. All *cyt b* haplotype sequences were deposited in GenBank under the accession number MK111987-MK111997 and MT013304-MT013306 (Additional file 2, Table S5).

### Genetic diversity, genetic differentiation and population structure

For each microsatellite locus the presence of null alleles was assessed using the software Microchecker v 2.2.3 [66]. Genetix v 4.05.2 [67] and FSTAT v 2.9.3.2 [68] were used to estimate the observed (H_o_) and the unbiased expected heterozygosity (H_e_), and the fixation coefficient (F_IS_) as an indicator for inbreeding. These estimates were obtained for all loci, for each site, and for each side of the river (Table 1). Hardy-Weinberg equilibrium (HWE) was tested for all loci and sites with 10,000 permutations using Genetix v 4.05.2. Allelic richness was calculated with FSTAT v 2.9.3.2 by calculating the standardised allelic richness for each locus and analysing the respective average per site and for each side of the river. Linkage disequilibrium (LD) among all pairs of loci was estimated using the correlation coefficient of Weir [69] and significant departures from LD were assessed by 10,000 permutations using Genetix v 4.05.2.

Pairwise genetic differentiation between forest sites was explored with Wright’s F-statistic according to Weir and Cockerham [70] implemented in Genetix v 4.05.2 with 10,000 permutations. Pairwise F_ST_-values between sites (with *n* ≥ 10) from the same riverside and from different riversides were assessed and compared to the overall set of pairwise comparisons. Conversely, interindividual relatedness was used as a measure of genetic similarity between forest sites. For this, the relatedness coefficient (*r*) of Queller and Goodnight [71] was calculated in Kingroup v 2.0 [72] for all possible pairwise comparisons. Mean relatedness was calculated for all dyads, and for the northern and southern subset separately. Between riverside comparisons were calculated, and within riversides within and among site comparisons were assessed for all forest sites with *n* ≥ 10. Significances between riversides (α = 5%) was tested for genetic differentiation and relatedness with a Mann-Whitney U Test implemented in *R* v 3.5.0 [73].

In order to investigate population genetic structure in *T. longipes* along the Kinabatangan River, Bayesian clustering was used to assign individuals to population clusters based on their genotypes without prior information on their sampling sites. The analysis was performed in *STRUCTURE* v 2.3.4 [74, 75] with *k* values ranging from 2 to 8. Each value of *k* was tested 20 independent times, under an admixture model with correlated allele frequencies between clusters and with each *STRUCTURE* run lasting 500,000 iterations of the Markov chain Monte Carlo (MCMC) algorithm discarding the first 20% steps as burn-in. To determine the most probable number of population clusters, the likelihood of the data (LnPr(k) [74];) and its rate of change (Δ*k* [38];) were inspected. For further examinations of potential sub-structuring along both riversides of the Kinabatangan River, individuals from the northern and southern riversides were analysed separately.

Genetic diversity within *T. longipes* was assessed by identifying mitochondrial haplotypes and by estimating haplotype (*h*) and nucleotide (*π*) diversity using DnaSP v 5.10.1 [76]. Haplotype genealogies were visualised with a minimum-spanning network computed in Arlequin v 3.5 [77].

### Migration rates

To estimate the recent migration rates between riversides and between forest sites within each riverside, a MCMC analysis was run for each dataset using the software BayesASS v 3 [78]. For each analysis, 10 million MCMC iterations were performed discarding the first 20% as burn-in. For each dataset three independent runs with different seed numbers were carried out, and convergence of the three runs was assessed by comparing the posterior estimates of each parameter (all three runs for each test gave the same results).

### Sex-biased dispersal

A possible sex-bias in dispersal was assessed by comparing male and female relatedness together with differences in male and female population assignment indices. The relatedness coefficient (*r*) of Queller and Goodnight [71] was calculated for all possible pairwise comparisons in Kingroup v 2.0 [72]. Mean relatedness was calculated for all male/male and female/female dyads and for males and females within and among sampling locations within riversides. The number of related dyads was determined for each sex. For each dyad the likelihood of a relationship of r ≥ 0.25 (parent – offspring, full siblings, aunt/uncle – nice/nephew, half siblings) was calculated in Kingroup against the null-hypothesis of being unrelated, estimated using 10,000 simulations from the allele frequency data. Dyads were classified as related when respective likelihood ratios reached α = 5% significance level. Furthermore, inter-individual spatial proximities of related dyads were assessed by measuring the closest (Euclidian) distance of two related individuals based on trapping data implemented in the *Animal Movement* extension in ArcView GIS 3.3 (ESRI, Inc.).

The relatedness analysis was complemented with corrected assignment index (*AIc*) of Goudet et al. [52]. The assignment index is centered on zero and gives the probability of an individual’s genotype occurring in the sampled sub-population compared to that by chance alone [52]. While negative *AIc* values indicate a genotype less likely than average to occur in the sampled population and characterises possible immigrants, a positive *AIc* value indicates probable residents. Therefore, the dispersing sex should be characterised by a lower mean *AIc* with a higher variance compared to the more philopatric sex [52]. Individual *AIc*s were calculated using the *R* package *hierfstat* v 0.04–22 [79] and mean *AIc* (*mAIc*) and variances (*vAIc*) were analysed for each sex separately. The two riversides were inspected separately to infer possible physical barriers on movements. A Mann-Whitney U Test was used to detect significant sex differences in relatedness and *mAIc*, and Levene’s test to detect differences between sexes in *vAIc*. All tests were performed using basic packages in R v 3.5.0 [73].

Finally, (matrilineal) mitochondrial markers represent another tool to infer sex-biased dispersal. Maternally related males and females share the same haplotypes, differences in haplotype diversity, the presence of sex-specific haplotypes and unique haplotypes within sampling locations (*haplotype singletons*), represent thus signals of immigration, and should be higher in the dispersing sex. Therefore, mitochondrial haplotype diversity was compared between males and females to infer sex-biased dispersal in *T. longipes* according to Brunke et al. [19].

## Supplementary information

**Additional file 1 Table S1** Amplification characteristics of single (S) and multiplex (M) reactions and the respective microsatellite loci used in *T. longipes*. **Table S2** Upper table: Genetic ([F_ST_-values], upper matrix) and geographic (Euclidean) distances ([km], bottom matrix) among forest sites. Lower table: Mean relatedness (*r*, upper matrix) and geographic (Euclidean) distances ([km], bottom matrix) among and within forest sites. **Table S3** Results from MWU test (Observed (*Ho*) and expected heterozygosity (*He*), inbreeding index (*F*_*IS*_), allelic richness, haplotype (*h*) and nucleotide (*π*) diversity, relatedness (*r*), on the northern and southern riverside, and relatedness (*r*), interindividual distances of related dyads (*r* ≥ 0.25), *mAIc* in males and females) or Levene’s test (*vAIc* in males and females). Tests were conducted for the whole sets and for within and among forest site/sampling location comparisons. Significant differences are in bold. **Table S4** Relative proportion of individuals per site and riverside, respectively, which were assigned to one of the clusters under *k* = 2 (LnP(D): − 3112.2), with a probability of q > 80%.

**Additional file 2 Table S5***Cytochrome b* haplotypes in *T. longipes* (*n* = 60) and their spatial distribution (upstream → downstream) along the northern (NA–ND) and southern (SE–SI) riverside. **Figure S1:** Haplotype network based on *cyt b* sequences.

**Additional file 3 Table S6** Home range area, the maximum (*D max*) and minimum (*D min*) home range diameter for the two *T. longipes* observed with the triangulation method. **Figure S2** Landscape changes along the Kinabatangan River between 1985 and time of study (2013). Study sites are marked with green lines (Pictures: Google Earth Pro V 7.3.2, 12/1985–12/2013, Kinabatangan River, lat.: 5.465153° long.: 118.071165°. [01/2020]).

## Data Availability

All data generated or analysed during this study are included in this published article (and its additional files). All *cyt b* haplotype sequences of *Tupaia longipes* were deposited in GenBank (Accession No.: MK111987-MK111997 and MT013304-MT013306).

## Abbreviations

CITES: Convention on International Trade in Endangered Species of Wild Fauna and Flora
cyt b: Cytochrome b
F_IS_: Inbreeding coefficient
F_ST_: Genetic differentiation between sub-populations
H_e_: Expected heterozygosity
H_o_: Observed heterozygosity
HWE: Hardy-Weinberg equilibrium
IBD: Isolation by distance
IUCN: International Union for Conservation of Nature
LD: Linkage disequilibrium
LKWS: Lower Kinabatangan Wildlife Sanctuary
mAIc: Mean of corrected assignment index
MCMC: Markov chain Monte Carlo
MWU Test: Mann-Whitney U Test
msats: Microsatellites
mtDNA: Mitochondrial DNA
vAIc: Variance of corrected assignment index
