# Supplementary material for: Dispersal and genetic structure in a tropical small mammal, the Bornean tree shrew (Tupaia longipes), in a fragmented landscape along the Kinabatangan River, Sabah, Malaysia
Source: BMC Genet. 2020 Apr 17;21:43. doi: 10.1186/s12863-020-00849-z (PMC7164274; doi:10.1186/s12863-020-00849-z)
Supplement: Supplementary file 1 — Additional file 1 Table S1 Amplification characteristics of single (S) and multiplex (M) reactions and the respective microsatellite loci used in T. longipes. Table S2 Upper table: Genetic ([FST-values], upper matrix) and geographic (Euclidean) distances ([km], bottom matrix) among forest sites. Lower table: Mean relatedness (r, upper matrix) and geographic (Euclidean) distances ([km], bottom matrix) among and within forest sites. Table S3 Results from MWU test (Observed (Ho) and expected heterozygosity (He), inbreeding index (FIS), allelic richness, haplotype (h) and nucleotide (π) diversity, relatedness (r), on the northern and southern riverside, and relatedness (r), interindividual distances of related dyads (r ≥ 0.25), mAIc in males and females) or Levene’s test (vAIc in males and females). Tests were conducted for the whole sets and for within and among forest site/sampling location comparisons. Significant differences are in bold. Table S4 Relative proportion of individuals per site and riverside, respectively, which were assigned to one of the clusters under k = 2 (LnP(D): − 3112.2), with a probability of q > 80%. [file 12863_2020_849_MOESM1_ESM.docx]

**Additional file 1**

**Table S1** Amplification characteristics of single (S) and multiplex (M) reactions and the respective microsatellite loci used in *T. longipes*

| Reaction | Annealing  temperature | Loci |
| --- | --- | --- |
| S1 | 65°C (-1°C) x 35 circles | Js22^a^ |
| S2 | 68°C (-1°C) x 9 circles,  58°C x 26 circles | TB14 ^b^ |
| M1 | 58°C x 35 circles | Js183 ^a^, TB18 ^b^ |
| M2 | 56°C x 35 circles | Js188 ^a^, TB8 ^b^ |
| M3 | 64°C (-1°C) x 5 circles,  58°C x 30 circles | TB15 ^b^, TB16 ^b^ |

^a^Munshi-South and Wilkinson 2006, ^b^Liu and Yao 2012

**Table S2** Upper table: Genetic ([F_ST_-values], upper matrix) and geographic (Euclidean) distances ([km], bottom matrix) among forest sites.

Lower table: Mean relatedness (*r*, upper matrix) and geographic (Euclidean) distances ([km], bottom matrix) among and within forest sites.

| *Distances [km]/F_ST_* | NA | NB | ND | SE | SF | SG |  |
| --- | --- | --- | --- | --- | --- | --- | --- |
| NA | - | 0.0158 | 0.0040 | 0.0438** | 0.0396** | 0.0475*** |  |
| NB | 9.71 | - | 0.0235** | 0.0544*** | 0.0569*** | 0.0497*** |  |
| ND | 22.36 | 15.51 | - | 0.0847*** | 0.0732*** | 0.0680*** |  |
| SE | 4.20 | 7.02 | 21.78 | - | 0.0239* | 0.0440*** |  |
| SF | 8.56 | 1.66 | 16.67 | 5.92 | - | 0.0381*** |  |
| SG | 13.58 | 4.23 | 11.61 | 11.58 | 5.74 | - |  |
|  |  |  |  |  |  |  |  |
| *Distance [km]/r* | NA | NB | ND | SE | SF | SG | Within sites |
| NA | - | -0.0256 | 0.0532 | -0.0323 | 0.0118 | -0.0092 | 0.0490 |
| NB | 9.71 | - | -0.0222 | -0.1027 | -0.0562 | -0.0577 | -0.0253 |
| ND | 22.36 | 15.51 | - | -0.1054 | -0.0463 | -0.0387 | 0.0712 |
| SE | 4.20 | 7.02 | 21.78 | - | 0.0294 | 0.0049 | 0.0640 |
| SF | 8.56 | 1.66 | 16.67 | 5.92 | - | 0.0488 | 0.1184 |
| SG | 13.58 | 4.23 | 11.61 | 11.58 | 5.74 | - | 0.1140 |

* ≤ 0.05, ** ≤ 0.01, *** ≤ 0.001

**Table S3** Results from MWU test (Observed (*Ho*) and expected heterozygosity (*He*), inbreeding index (*F_IS_*), allelic richness, haplotype (*h*) and nucleotide (*π*) diversity, relatedness (*r*), on the northern and southern riverside, and relatedness (*r*), interindividual distances of related dyads (*r* ≥ 0.25), *mAIc* in males and females) or Levene’s test (*vAIc* in males and females). Tests were conducted for the whole sets and for within and among forest site/sampling location comparisons. Significant differences are in bold

| North *vs* South | ALL |  | Within forest sites |  | Among forest sites |  |
| --- | --- | --- | --- | --- | --- | --- |
| MWU Test | Z | p | Z | p | Z | p |
| *Ho* | 1.5972 | 0.110 |  |  |  |  |
| *He* | 1.4434 | 0.149 |  |  |  |  |
| *F_IS_* | -0.2887 | 0.773 |  |  |  |  |
| *allelic richness* | 2.3094 | **0.021** |  |  |  |  |
| *h* | 1.4434 | 0.149 |  |  |  |  |
| *π* | 1.3229 | 0.186 |  |  |  |  |
| *F_ST_* | -1.9640 | **0.050** |  |  |  |  |
| *r* | -7.2962 | **< 0.001** | -6.0751 | **< 0.001** | -4.1245 | **< 0.001** |
|  |  |  |  |  |  |  |
| Males *vs* Females | ALL |  | Within sampling locations |  | Among sampling locations |  |
| MWU Test | Z | p | Z | p | Z | p |
| *r* | -3.1440 | **0.002** | 0.4100 | 0.682 | -3.0987 | **0.002** |
| distance among  related dyads | -2.9150 | **0.004** | 0.0704 | 0.944 | -3.2379 | **0.0012** |
| *mAIc* | -0.0618 | 0.953 |  |  |  |  |
|  |  |  |  |  |  |  |
| Males *vs* Females | ALL |  |  |  |  |  |
| Levene’s Test | F | p |  |  |  |  |
| *vAIc* | 9.6273 | **0.002** |  |  |  |  |

**Table S4** Relative proportion of individuals per site and riverside, respectively, which were assigned to one of the clusters under *k* = 2 (LnP(D): -3112.2), with a probability of q > 80%

|  | Cluster I  (n = 60) [%] | Cluster II  (n = 43) [%] |
| --- | --- | --- |
| NA | 81.82 | 9.09 |
| NB | 100.00 | 0.00 |
| NC | 100.00 | 0.00 |
| ND | 94.12 | 0.00 |
| *North* | *94.12* | *1.96* |
| SE | 9.09 | 81.82 |
| SF | 0.00 | 80.95 |
| SG | 27.59 | 55.17 |
| SI | 27.59 | 55.17 |
| *South* | *18.46* | *64.62* |
