# Supplementary material for: Dispersal and genetic structure in a tropical small mammal, the Bornean tree shrew (Tupaia longipes), in a fragmented landscape along the Kinabatangan River, Sabah, Malaysia
Source: BMC Genet. 2020 Apr 17;21:43. doi: 10.1186/s12863-020-00849-z (PMC7164274; doi:10.1186/s12863-020-00849-z)
Supplement: Supplementary file 2 — Additional file 2 Table S5Cytochrome b haplotypes in T. longipes (n = 60) and their spatial distribution (upstream → downstream) along the northern (NA–ND) and southern (SE–SI) riverside. Figure S1: Haplotype network based on cyt b sequences. [file 12863_2020_849_MOESM2_ESM.docx]

**Additional file 2**

**Table S5** *Cytochrome b* haplotypes in *T. longipes* (n = 60) and their spatial distribution (upstream → downstream) along the northern (NA – ND) and southern (SE – SI) riverside

|  | Haplotype frequency |  |  |  |  |  |  |  |  |  |  |  |  |  |  |  |  |
| --- | --- | --- | --- | --- | --- | --- | --- | --- | --- | --- | --- | --- | --- | --- | --- | --- | --- |
| Haplotype | Total | NA_1_ | NA_2_ | NB_1_ | NB_2_ | NC_1_ | NC_4_ | ND_1_ | ND_2_ | ND_3_ | SE_1_ | SE_2_ | S_F_ | SG_1_ | SG_2_ | SI | Accession No. |
| Tl 1 | 13 | - | - | - | - | 1 | - | - | - | - | 1 | 1 | 3 | 5 | 2 | - | MK111987 |
| Tl 2 | 2 | - | - | - | - | - | - | - | - | - | - | - | - | - | - | 2 | MK111988 |
| Tl 3 | 3 | 2 | 1 | - | - | - | - | - | - | - | - | - | - | - | - | - | MK111989 |
| Tl 4 | 2 | - | 1 | 1 | - | - | - | - | - | - | - | - | - | - | - | - | MK111990 |
| Tl 5 | 2 | 1 | - | 1 | - | - | - | - | - | - | - | - | - | - | - | - | MK111991 |
| Tl 6 | 14 | - | - | - | - | - | - | - | - | - | 2 | 1 | 2 | 8 | 1 | - | MK111992 |
| Tl 7 | 7 | 1 | - | - | 1 | - | 1 | 1 | 1 | 2 | - | - | - | - | - | - | MK111993 |
| Tl 8 | 2 | - | - | - | - | - | - | - | 1 | - | - | - | - | - | - | 1 | MK111994 |
| Tl 9 | 4 | - | - | - | - | - | - | 2 | - | 1 | - | - | 1 | - | - | - | MK111995 |
| Tl 10 | 1 | 1 | - | - | - | - | - | - | - | - | - | - | - | - | - | - | MK111996 |
| Tl 11 | 5 | - | - | 3 | 1 | - | - | - | - | 1 | - | - | - | - | - | - | MK111997 |
| Tl 12 | 1 | - | - | - | - | - | - | 1 | - | - | - | - | - | - | - | - | MT013304 |
| Tl 13 | 1 | - | 1 | - | - | - | - | - | - | - | - | - | - | - | - | - | MT013305 |
| Tl 14 | 3 |  | - | - | - | - | - | - | - | - | - | 1 | 1 | - | 1 | - | MT013306 |


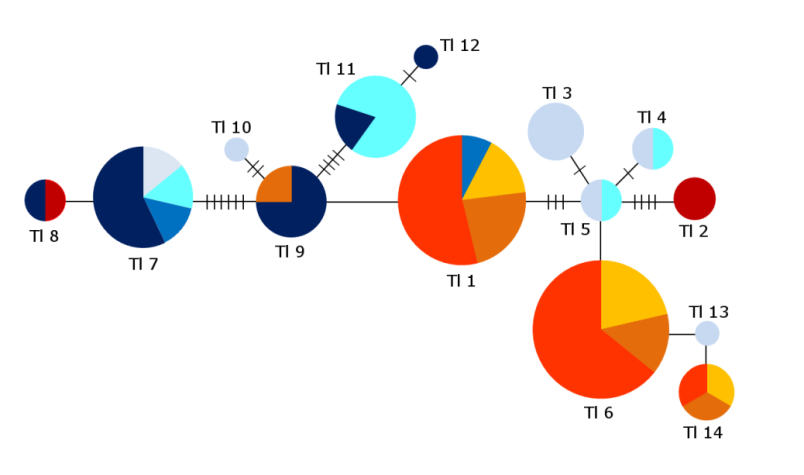

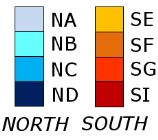


**Figure S1:** Haplotype network based on *cyt b* sequences
