# Supplementary material for: Dispersal and genetic structure in a tropical small mammal, the Bornean tree shrew (Tupaia longipes), in a fragmented landscape along the Kinabatangan River, Sabah, Malaysia
Source: BMC Genet. 2020 Apr 17;21:43. doi: 10.1186/s12863-020-00849-z (PMC7164274; doi:10.1186/s12863-020-00849-z)
Supplement: Supplementary file 3 — Additional file 3 Table S6 Home range area, the maximum (D max) and minimum (D min) home range diameter for the two T. longipes observed with the triangulation method. Figure S2 Landscape changes along the Kinabatangan River between 1985 and time of study (2013). Study sites are marked with green lines (Pictures: Google Earth Pro V 7.3.2, 12/1985–12/2013, Kinabatangan River, lat.: 5.465153° long.: 118.071165°. [01/2020]). [file 12863_2020_849_MOESM3_ESM.docx]

**Additional file 3**

**Table S6** Home range area, the maximum (*D max*) and minimum (*D min*) home range diameter for the two *T. longipes* observed with the triangulation method

| Sex | Number of individuals | Area [ha] | D max [m] | D min [m] |
| --- | --- | --- | --- | --- |
| Male | 1 | 15.6 | 621.43 | 305.86 |
| Female | 1 | 13.4 | 675.71 | 264.23 |

**2013**

**2000**


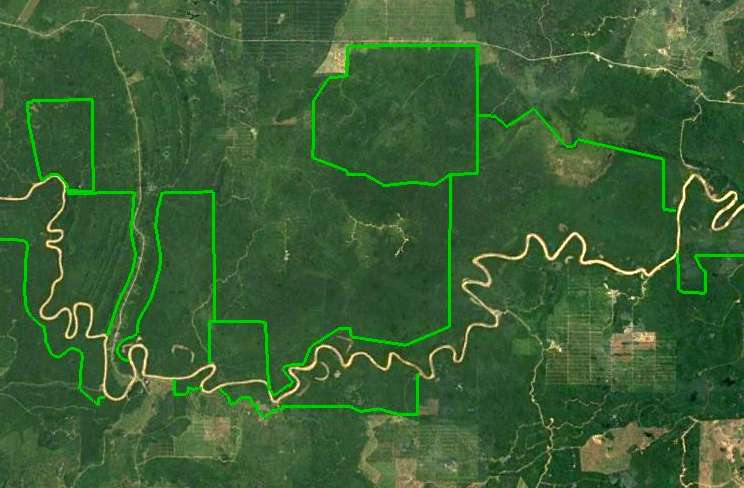


**1990**


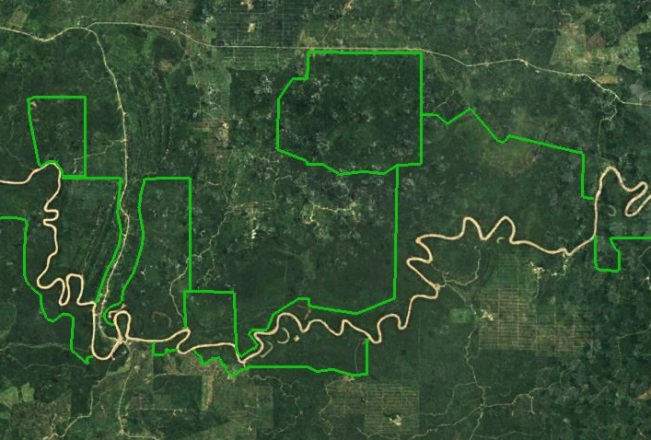


**1985**


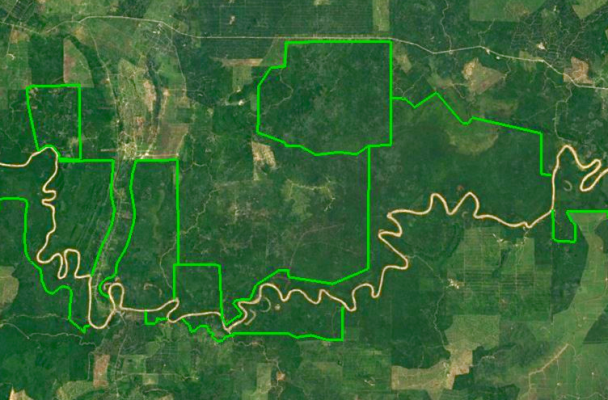


**1995**


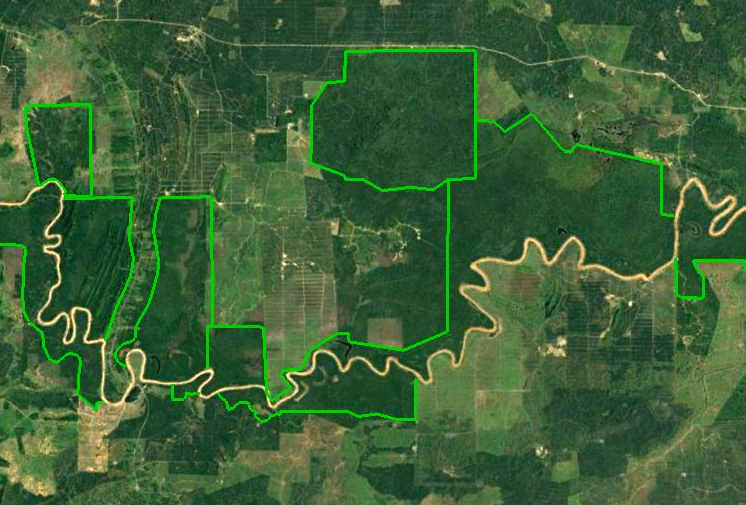


**2000**


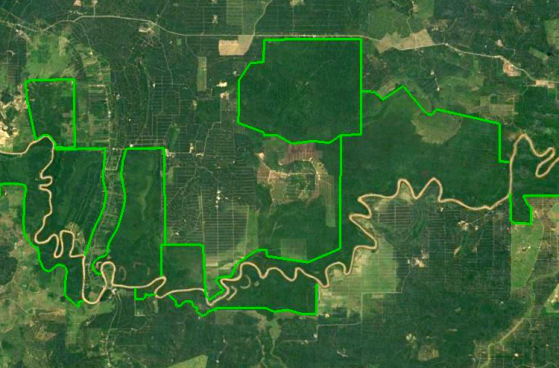


**2005**


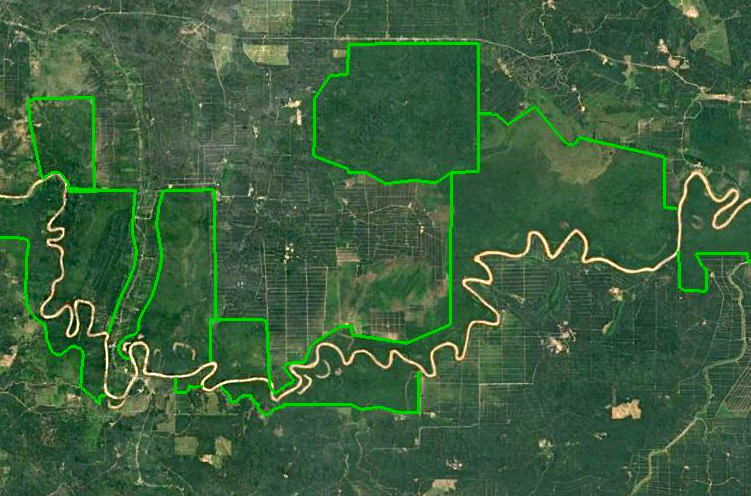


**2013**

**Figure S2** Landscape changes along the Kinabatangan River between 1985 and time of study (2013). Study sites are marked with green lines (Pictures: Google Earth Pro V 7.3.2, 12/1985 – 12/2013, Kinabatangan River, lat.: 5.465153° long.: 118.071165°. [01/2020])
